# Cyclical Exacerbation of Asthma Symptoms in Relation to the Menstrual Cycle: A Case Report on Catamenial Asthma

**DOI:** 10.7759/cureus.64512

**Published:** 2024-07-14

**Authors:** Gurjot Singh, Shubam Trehan, Kanishka Goswami, Adarshpreet Singh, Piyush Puri

**Affiliations:** 1 Internal Medicine, Maharaj Sawan Singh Charitable Hospital, Beas, IND; 2 Medicine, Punjab Institute of Medical Sciences, Jalandhar, IND

**Keywords:** asthma management, case report, spirometry, hormonal fluctuations, menstruation, catamenial asthma

## Abstract

Catamenial asthma, marked by cyclical exacerbations of symptoms linked to the menstrual cycle, poses distinctive diagnostic and therapeutic challenges. This report discusses a 34-year-old woman who experienced significant asthma flare-ups 3-5 days before menstruation, as confirmed by spirometry (forced expiratory volume in one second (FEV1) dropped from 2.5 to 1.75 liters). Despite adhering to standard asthma treatments, her symptoms remained poorly controlled during these periods. A comprehensive management plan encompassing inhaled corticosteroids, short-acting beta-agonists, montelukast, and oral contraceptives, along with lifestyle modifications and patient education, led to a significant improvement in FEV1 and reduced symptom severity. This case underscores the need for personalized treatment strategies that take hormonal influences into account, suggesting that integrating hormonal therapies with conventional asthma management can yield significant benefits.

## Introduction

Catamenial asthma, a subtype of asthma characterized by the worsening of symptoms in relation to the menstrual cycle, represents a significant yet often under-recognized clinical phenomenon. This condition underscores the complex interplay between sex hormones and respiratory health, posing unique challenges in diagnosis and management. Understanding the pathogenesis, epidemiology, clinical features, and treatment of catamenial asthma is crucial for optimizing patient outcomes [1].

Previous research indicates a connection between hormonal fluctuations and respiratory function. For instance, studies have shown that women often experience asthma exacerbations during specific phases of their menstrual cycle, highlighting the need for heightened awareness among clinicians [1].

The pathogenesis of catamenial asthma is primarily driven by hormonal fluctuations throughout the menstrual cycle. Estrogen and progesterone, the key hormones in the female reproductive system, influence various physiological processes, including immune function and airway responsiveness. Estrogen, which rises during the follicular phase, enhances immune responses and increases airway inflammation through the upregulation of inflammatory mediators like interleukins and tumor necrosis factor-alpha (TNF-α) [2]. Progesterone, which peaks during the luteal phase, has both pro-inflammatory and anti-inflammatory effects. High levels of progesterone may exacerbate asthma symptoms by enhancing bronchial hyperreactivity [3]. The decline in both estrogen and progesterone levels just before menstruation creates a hormonal environment conducive to asthma exacerbations, with a relative increase in testosterone further contributing to airway reactivity [4].

Recent studies further elucidate these mechanisms, showing how hormonal receptors in the airway smooth muscle can alter bronchial reactivity and immune cell function during different menstrual phases [5].

Catamenial asthma affects a significant proportion of women with asthma. Studies suggest that up to 30-40% of women with asthma experience symptom exacerbation related to their menstrual cycle [6]. This subtype is most commonly observed in women aged 20-45 years, coinciding with their reproductive years. The exact prevalence varies widely across different populations, influenced by genetic, environmental, and lifestyle factors. For instance, certain populations may exhibit higher prevalence due to genetic predispositions or environmental triggers [5].

This case report delves into the clinical presentation and spirometry findings of a patient with catamenial asthma, focusing on analyzing the menstrual changes contributing to her condition. By examining this specific case, we aim to elucidate the complex hormonal dynamics underlying catamenial asthma, thereby guiding the development of more precise and effective treatment options for women affected by this condition.

## Case presentation

A 34-year-old female day-time office worker with a body mass index (BMI) of 23.4 kg/m² presented to a tertiary care hospital with a history of recurrent asthma exacerbations. She had been experiencing these exacerbations, specifically 3-5 days prior to the onset of her menstrual period, with symptoms subsiding shortly after menstruation. The patient was diagnosed with mild asthma at the age of 25. She also had a history of seasonal allergies but no significant dermatologic history, no past history of COVID-19, no history of active or passive smoking and drug addiction, and a history of occasional alcohol intake. Her family history revealed that her mother had mild asthma, but there were no other significant respiratory conditions in the family. Her asthma had been well-controlled with her regular medication regimen until about six months before her presentation.

Approximately six months ago, the patient began experiencing increased asthma symptoms correlating with her menstrual cycle. She reported that her symptoms, including wheezing, shortness of breath, and chest tightness, would significantly worsen 3-5 days before her period and would only improve a few days after her period began. This cyclical exacerbation of symptoms was having a substantial impact on her daily activities and work performance. She described her symptoms during these periods as "overwhelming," stating that she often had to take time off work due to severe shortness of breath and chest tightness. Despite using her inhaler more frequently, she found little relief during the premenstrual and menstrual phases.

The patient was adhering to her prescribed asthma medication regimen, which included inhaled corticosteroids (ICS) (fluticasone) and a short-acting beta-agonist (SABA) (albuterol) as needed. Despite this, she found her symptoms were not well-controlled during the premenstrual and menstrual phases. A respiratory examination revealed bilateral wheezing, more pronounced in the lower lung fields, without the use of accessory muscles for breathing. Lab reports came normal (Table 1).

**Table 1:** Lab reports

Lab reports	Results	Reference range
White blood cell count (1000/cmm)	9.4	4-11
Platelet count (1000/cmm)	191	150-450
Red blood cell count (million/uL)	4.89	4.0-5.1
Hemoglobin (g/dL)	11.5	12-14
Mean corpuscular volume (fL)	78.4	80-100
Mean corpuscular hemoglobin (pg)	27.5	27.5-33.2
Mean corpuscular hemoglobin concentration (gm/dL)	33.4	33.4-35.5
Absolute eosinophil count (1000/cmm)	450.91	20-500
Neutrophils (%)	80	40-80
Lymphocytes (%)	10	20-40
Eosinophils (%)	06	1-6
Monocytes (%)	04	2-10
Basophils (%)	00	0-1
Erythrocyte sedimentation rate (mm/hr)	2	0-20
C-reactive protein (mg/dL)	0.2	<0.3
IgE antibodies (mg/dL)	0.1	<0.3

Allergy testing was negative for common allergens, ruling out allergic triggers as a primary cause. Further, a chest X-ray showed no signs of infection or other pulmonary pathology.

**Figure 1:**
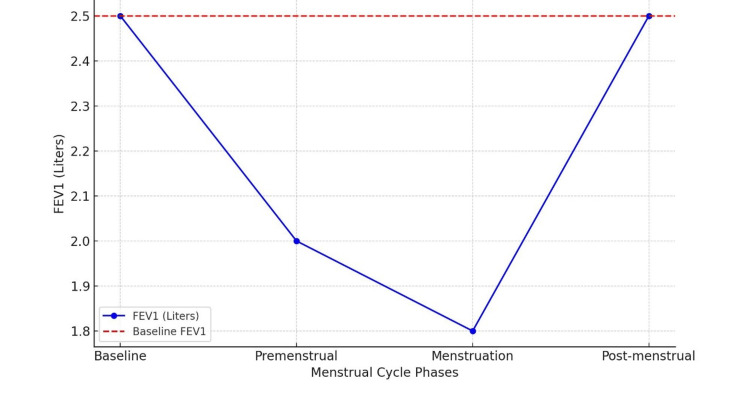
Graphical presentation of spirometry changes in catamenial asthma The spirometry chart illustrates the FEV1 across different phases of the menstrual cycle. The X-axis represents the timeline from premenstrual, through menstrual, to postmenstrual, while the Y-axis shows FEV1 values in liters. Data points indicate significant FEV1 drops from 2.5 to 1.75 liters during premenstrual and menstrual phases, followed by a gradual return to baseline postmenstruation. This pattern highlights the cyclical nature of catamenial asthma. FEV1: forced expiratory volume in one second

Spirometry was performed to assess lung function during different phases of her menstrual cycle (Table 2). The results indicated a significant decline in forced expiratory volume in one second (FEV1) during the premenstrual and menstrual phases (Figure 1). These findings correlated with her symptomatic periods, confirming airflow obstruction during these phases.

**Table 2:** Spirometry report conducted in different phases of menstrual cycle Baseline phase: normal lung function with an FEV1 of 2.5 liters and an FEV1/FVC ratio of 0.80. Premenstrual phase (3-5 days before menses): mild obstruction with an FEV1 decrease of approximately 20% from baseline (2.0 liters, FEV1/FVC ratio 0.75). During menstruation: moderate obstruction with an FEV1 decrease of 30% from baseline (1.75 liters, FEV1/FVC ratio 0.70). Postmenstrual phase (one week after menses): return to normal lung function with FEV1 back to baseline levels (2.5 liters, FEV1/FVC ratio 0.80). FEV1: forced expiratory volume in one second; FVC: forced vital capacity

Phase	FEV1 (liters)	FEV1/FVC ratio
Baseline	2.50	0.80
Premenstrual	2.00	0.75
Menstruation	1.75	0.70
Postmenstrual	2.50	0.80

Based on the cyclical pattern of asthma exacerbations correlating with the patient's menstrual cycle, supported by spirometry findings, the diagnosis of catamenial asthma was established by ruling out all other possible diagnosis. The patient was advised to continue her inhaled corticosteroid (fluticasone) twice daily to maintain baseline asthma control and use her SABA (albuterol) as needed, with increased monitoring during premenstrual and menstrual phases. She was also prescribed montelukast 10 mg nightly to reduce inflammation and bronchoconstriction. Oral contraceptives (norgestrel 0.3 mg + ethinyl estradiol 30 µg) were added to the treatment plan. After three months of follow-up, she had shown significant improvement in premenstrual and menstrual asthma exacerbations. 

## Discussion

Catamenial asthma is a unique subtype of asthma that exhibits symptom exacerbations closely aligned with the menstrual cycle. This case report illustrates the complex interplay between menstrual cycle changes and asthma control, emphasizing the importance of recognizing and managing this condition effectively.

The pathogenesis of catamenial asthma is intricately linked to the cyclical changes in estrogen and progesterone levels during the menstrual cycle. Estrogen, primarily elevated during the follicular phase, has been shown to enhance immune responses and increase airway inflammation. This can be attributed to estrogen's ability to upregulate the expression of inflammatory mediators such as interleukins and TNF-α, which contribute to airway hyperresponsiveness [2]. Additionally, estrogen influences the expression of beta-adrenergic receptors in the airway smooth muscle, potentially affecting bronchial reactivity [5]. Progesterone, which peaks during the luteal phase, exerts a modulatory effect on the immune system. It has both pro-inflammatory and anti-inflammatory effects, making its role in catamenial asthma complex. High levels of progesterone may exacerbate asthma symptoms by enhancing bronchial hyperreactivity [3]. Progesterone also affects the production of prostaglandins, which can modulate airway tone and reactivity. The decline in both estrogen and progesterone levels just before menstruation creates a hormonal environment conducive to asthma exacerbations. The relative increase in testosterone during this phase may further contribute to airway reactivity [4].

Studies have shown that the menstrual cycle's hormonal fluctuations can significantly impact asthma control. For example, a study by Pinkerton et al. [7] found that asthma symptoms and peak expiratory flow rates were significantly worse in the late luteal and early follicular phases compared to other phases of the menstrual cycle. This finding underscores the need for heightened awareness and proactive management during these critical periods.

Catamenial asthma affects a significant proportion of women with asthma. Studies suggest that up to 30-40% of women with asthma experience symptom exacerbations related to their menstrual cycle [6]. This subtype is most commonly observed in women aged 20-45 years, coinciding with their reproductive years [7]. The exact prevalence varies widely across different populations, influenced by genetic, environmental, and lifestyle factors. The cyclical nature of catamenial asthma presents unique clinical challenges. Symptoms such as wheezing, shortness of breath, and chest tightness typically worsen during the luteal phase and menstruation. This pattern was clearly observed in our patient, who experienced the most severe asthma exacerbations just before her period, necessitating increased use of her rescue inhaler.

Diagnosing catamenial asthma requires a thorough clinical evaluation to correlate asthma symptoms with the menstrual cycle. Key diagnostic steps include obtaining a detailed menstrual history and performing spirometry at different phases of the menstrual cycle to detect cyclical changes in lung function. In our case, spirometry revealed significant declines in FEV1 during the premenstrual phase, with further decreases during menstruation. These findings correlated with the patient's symptomatic periods, confirming airflow obstruction during these phases. Allergy testing and chest imaging were also performed to rule out other potential triggers and comorbidities. A study by Kocurek and Hemnes [8] highlighted the importance of cyclical spirometry and hormonal measurements in diagnosing catamenial asthma. The study showed that patients with catamenial asthma exhibited significant cyclical changes in lung function, aligning with hormonal fluctuations.

Effective management of catamenial asthma requires a multifaceted approach that addresses both the hormonal and inflammatory components of the disease. Pharmacologic treatments, including ICS and SABA, are essential for maintaining baseline asthma control and managing acute exacerbations. In our case, the patient was advised to continue her ICS (fluticasone) and use her SABA (albuterol) as needed, with increased monitoring during premenstrual and menstrual phases. Leukotriene receptor antagonists, such as montelukast, can be beneficial in reducing inflammation and bronchoconstriction. Oral contraceptives were also prescribed, which had shown some improvement in symptoms. In refractory cases, gonadotropin-releasing hormone (GnRH) agonists may be used to suppress ovarian hormone production, thereby minimizing symptom variability [3]. A study by Rodriguez Bauza and Silveyra [9] demonstrated that hormonal therapies, including oral contraceptives, could significantly reduce asthma exacerbations in women with catamenial asthma. This supports the integration of hormonal treatments into the management plan for such patients.

Lifestyle modifications play a crucial role in managing catamenial asthma. Weight management through diet and exercise can help mitigate asthma severity, as obesity is a known risk factor for asthma. Stress management techniques, such as mindfulness and relaxation exercises, can alleviate symptoms exacerbated by hormonal fluctuations. Stress is known to impact hormonal balance and immune function, potentially influencing asthma control. Educating patients about the potential impact of hormonal changes on asthma control and providing resources for managing symptoms and understanding triggers can empower patients to take an active role in their care. Encouraging adherence to prescribed therapies and regular follow-up appointments can improve long-term outcomes. A longitudinal study by Thomas and Tenholder [10] emphasized the importance of regular monitoring and patient education in managing catamenial asthma. The study found that patients who received comprehensive education and regular follow-up had better asthma control and fewer exacerbations.

Ongoing research is needed to further elucidate the mechanisms driving catamenial asthma and develop targeted therapeutic strategies. Understanding the molecular and cellular pathways involved in hormonal modulation of airway reactivity can provide insights into novel treatment approaches. Additionally, investigating the role of other factors, such as genetic predisposition and environmental triggers, can enhance our understanding of this complex condition. Personalized medicine approaches that consider individual hormonal profiles and responses to treatment may improve management strategies for catamenial asthma. Integrating hormonal therapies with conventional asthma treatments can significantly improve outcomes for women affected by this condition. A recent study by Ensom [11] explored the potential of precision medicine in treating hormone-related respiratory disorders, highlighting the need for individualized treatment plans based on hormonal and genetic profiles.

## Conclusions

Catamenial asthma exemplifies the intricate relationship between menstrual changes and respiratory health. Understanding its pathogenesis, epidemiology, and clinical features is essential for effective diagnosis and management. This case report highlights the importance of recognizing hormonal influences in patients with asthma and tailoring treatment plans accordingly. Integrating hormonal therapies with conventional asthma management strategies can significantly improve outcomes for women affected by this condition. Ongoing research and awareness are essential to advance our understanding of catamenial asthma and optimize care for affected patients.
